# Effect of sow feed intake on litter growth and sow body weight change from farrowing to weaning

**DOI:** 10.1093/tas/txag140

**Published:** 2026-09-16

**Authors:** Mikayla S Spinler, Jason C Woodworth, Mike D Tokach, Robert D Goodband, Joel M DeRouchey, Katelyn N Gaffield, Jordan T Gebhardt

**Affiliations:** Department of Animal Sciences and Industry, College of Agriculture, Kansas State University, Manhattan, KS 66506, United States; Department of Animal Sciences and Industry, College of Agriculture, Kansas State University, Manhattan, KS 66506, United States; Department of Animal Sciences and Industry, College of Agriculture, Kansas State University, Manhattan, KS 66506, United States; Department of Animal Sciences and Industry, College of Agriculture, Kansas State University, Manhattan, KS 66506, United States; Department of Animal Sciences and Industry, College of Agriculture, Kansas State University, Manhattan, KS 66506, United States; Department of Animal Sciences and Industry, College of Agriculture, Kansas State University, Manhattan, KS 66506, United States; Department of Diagnostic Medicine/Pathobiology, College of Veterinary Medicine, Kansas State University, Manhattan, KS 66506, United States

**Keywords:** feed intake, gilts, lactation, litter performance, sows

## Abstract

A retrospective analysis was conducted to determine the relationship between lactation average daily feed intake (*ADFI*) and litter average daily gain (*ADG*) and sow body weight (*BW*) change over lactation. Data from 991 gilts and 3393 multiparous sows across eight studies were used. Gilts and multiparous sow ADFI were categorized into quintiles, and linear and quadratic orthogonal polynomial contrasts were tested to determine the relationship between lactation ADFI and sow and litter performance. Gilts lost less weight (quadratic, *P* < 0.05) from farrow to weaning as lactation feed intake increased with the largest change in BW loss occurring between gilts with an ADFI of* ≤ *5.30 and between 5.30 and 5.90 kg/d. In multiparous sows, BW change from farrow to weaning shifted from weight loss to gain (linear, *P* < 0.05) with increasing ADFI. Gilts had greater body weight loss during lactation compared to multiparous sows, with over half of the multiparous sows in the analysis gaining body weight. In both gilts and multiparous sows, increasing ADFI increased (linear; *P* < 0.05) litter ADG as well as litter size at weaning. Regression equations were developed to predict litter ADG and sow BW change for gilts and multiparous sows over lactation using backward stepwise regression. Lactation ADFI and litter ADG accounted for over half of the variation in maternal BW change over lactation, and litter size at weaning accounted for over half of the variation in litter ADG. Regression equations indicate that a one kg increase in ADFI increased litter ADG by 107 and 60 g and decreased BW loss by 4.74 and 5.19 kg for gilts and multiparous sows, respectively, when all other variables are held constant. At similar ADFI levels, multiparous sows may lose, maintain, or gain body weight during lactation. Sows that have both high feed intake and lose BW during lactation have greater litter ADG compared to sows with similar feed intake that gain BW during lactation. The analysis indicates that while increased feed intake during lactation improves both litter growth and reduces sow BW change, ADFI does not fully explain the variation in performance. The analysis indicates that over half of the multiparous sows gain BW from farrow to weaning, while over 80% of gilts lose BW. For both gilts and multiparous sows, gilts and sows with similar ADFI that lose BW over lactation have greater litter ADG compared to those that gain BW during lactation.

## Teaser

Lactation feed intake, sow body weight change, and litter growth performance are closely related, with high feed intake decreasing sow body weight loss, and higher litter growth increasing sow body weight loss. Overall, sows with high lactation feed intake that lose body weight over lactation have higher litter average daily gain compared to sows with similar feed intake that gain body weight.

## Introduction

Because lactation feed intake affects litter growth, sow body condition, and subsequent reproductive performance (Eissen et al. 2003), the goal of feeding sows during lactation is to maximize feed intake for milk production and reduce mobilization of body reserves (Tokach et al. 2019). When feed intake during lactation does not meet a sow’s nutrient requirements, sows will mobilize body reserves to compensate for deficient nutrient intake (Clowes et al. 2003). Gourley et al. (2017) observed that at deficient Lys intake levels, sows mobilized body reserves to support litter growth, resulting in no differences in litter growth rate. However, high body weight (*BW*) loss can have negative consequences on subsequent reproductive performance. Multiple studies have observed that an increase in sow BW loss during lactation leads to an increase in wean-to-service interval (*WSI*; Eissen et al. 2003; Thaker and Bilkei 2005; Strathe et al. 2017). However, recent research indicates that sows are not mobilizing body reserves even when they are deficient in Lys or other nutrients, resulting in lower litter growth performance (Liu et al. 2020; Spinler et al. 2024, 2025). However, contrary to previous findings indicating that sows mobilize body reserves when nutrient intake is insufficient (Revell et al. 1998; Eissen et al. 2003; Costermans et al. 2020; Muller et al. 2022), modern sows are not mobilizing body reserves in response to inadequate nutrient intake. Potential reasons include high lactation feed intake, resulting in decreased needs to mobilize body reserves, and improvements in genetics leading to leaner, more efficient sows with a lower basal metabolic requirement.

There is a relationship between feed intake, milk production, and litter growth performance, as an increase in feed intake, and therefore energy, supports milk yield and, consequently, improves litter growth (Strathe et al. 2017; Hawe et al. 2020; Estrada et al. 2024). Strathe et al. (2017) developed a regression equation and determined that a one kg increase in average daily feed intake (*ADFI*) can increase litter average daily gain (*ADG*) by 220 to 440 g/d, depending on sow parity. Individual amino acids (*AA*) and energy intake, not just total feed intake, can affect milk yield and milk composition (Theil et al. 2014). Therefore, ensuring sows consume adequate feed during lactation is critical for meeting both energy and AA requirements and supporting optimal litter growth.

Sow lactation feed intake has increased by roughly 30% over the last 30 years (Rodríguez et al. 2023), leading to increased sow productivity. Sows classified as having a rapid increase in ADFI over lactation, characterized by an early peak before day 10 of lactation with no subsequent decline, averaged 5.87 kg of feed intake (Koketsu et al. 1996b). In contrast, sows classified as having a rapid increase in ADFI using the same characterization by Estrada et al. (2024) had an ADFI of 7.90 kg, a 25% increase compared to Koketsu et al. (1996b). Given the high feed intake levels currently observed in commercial herds, it remains uncertain whether the relationship between ADFI, litter ADG, and sow BW change during lactation is linear or if a breakpoint exists beyond which additional intake no longer benefits sow or litter performance. High lactation feed intake may exceed nutrient requirements for maintenance and maximizing litter ADG for some sows, leading to sow weight gain and increased nutrient excretion. Due to recent increases in sow feed intake, higher litter growth, and variations in how sows mobilize body reserves in response to low nutrient intake, the objective of this retrospective analysis was to quantify the relationship between lactation ADFI, litter ADG, and sow BW change over lactation. We hypothesized that there would be a quadratic relationship between ADFI and sow BW change and litter ADG, where increases in feed intake beyond a certain point would no longer improve sow or litter performance.

## Materials and methods

The initial analysis included data from eight different trials totaling 4516 gilts and sows. Research trials were conducted in Iowa, Kansas, and Minnesota, USA, and Canada from 2018 to 2023. Trials were conducted across four research facilities, including both university and commercial research environments. Data from individual research trials were included in the analysis that recorded individual sow lactation feed intake, sow body weight at entry into farrowing and at weaning, litter weight at d 2, after litter equalization, and at weaning, as well as provided complete diet formulations. Individual sow and litter data were recorded for each trial. For the following reasons, 132 gilts and multiparous sows were removed: missing feed intake data, ADFI below 2.7 kg, ADFI above 11.3 kg, litter size at weaning was greater than litter size at d 2, mortality greater than 7 pigs, and maternal weight gain over lactation greater than 90 kg. The final dataset included totaled 4384 females, of which there were 991 gilts and 3393 multiparous sows (Table S1, available as supplementary data at *Translational Animal Science* online). In the current analysis, gilts are defined as animals that are nursing their first litter. Tables 1 and 2 describe the range in performance for gilts and multiparous sows across the eight trials, as well as the range in formulated dietary net energy (NE) levels and Lys:AA ratios.

**Table 1:** Descriptive statistics of data used in the analysis—gilts.

	Minimum	Average	Maximum
**NE, kcal/kg**	2401	2441	2507
**SID Lys**	0.59	1.02	1.28
**Met:Lys**	0.26	0.30	0.50
**Trp:Lys**	0.18	0.19	0.28
**Thr:Lys**	0.62	0.64	0.95
**Val:Lys**	0.70	0.74	1.27
**ADFI, kg**	2.8	6.2	9.5
**Gilt BW**			
**Entry, kg**	147.0	219.5	333.5
**Farrow, kg**	131.0	198.6	304.8
**Weaning, kg**	125.6	193.4	307.5
**Pig count, d 2**	10.0	15.0	19.0
**Pig count weaning**	7.0	13.4	18.0
**Litter weight, d 2, kg**	10.0	21.4	38.4
**Litter weight, weaning, kg**	30.3	70.8	114.0
**Pig weight, d 2, kg**	0.7	1.4	2.7
**Pig weight, weaning, kg**	2.6	5.3	9.5

**Table 2:** Descriptive statistics of data used in the analysis—multiparous sows.

	Minimum	Average	Maximum
**NE, kcal/kg**	2408	2401	2507
**SID Lys**	0.58	1.03	1.28
**Met:Lys**	0.30	0.26	0.45
**Trp:Lys**	0.16	0.19	0.26
**Thr:Lys**	0.52	0.64	0.87
**Val:Lys**	0.69	0.69	1.15
**ADFI, kg**	2.7	7.2	11.3
**Sow BW**			
**Entry, kg**	155.6	276.9	404.0
**Farrow, kg**	140.5	276.9	381.4
**Weaning, kg**	141.5	257.4	386.0
**Pig count d 2**	7.0	14.5	20.0
**Pig count weaning**	6.0	12.5	20.0
**Litter weight d 2, kg**	9.8	22.2	38.5
**Litter weight weaning, kg**	30.6	76.6	129.5
**Pig weight d 2, kg**	0.7	1.5	2.7
**Pig weight weaning, kg**	3.2	6.1	10.9

For all trials, sows were weighed at entry into farrowing and before weaning. Two trials recorded sow BW after farrowing. For trials that did not record sow BW after farrowing, the equation developed by Mallmann et al. (2018) was used to estimate sow BW after farrowing. Litter size was recorded at d 2 and at weaning. Day 2 litter size refers to the number of pigs in each litter after pigs were cross-fostered to equalize litter size. Litter weight was recorded after litters were equalized and at weaning. Sow lactation feed intake was recorded using electronic feeders (Jyga Technologies, St-Lambert-de-Lauzon, Quebec, CA). Sows were provided ad libitum access to feed in all studies. Diets for each experimental treatment within a trial were reformulated using NRC (2012) nutrient values for ingredients to standardize nutrient concentrations. These values were then used to determine average NE, kcal/d, and AA, g/d, intake over lactation based on lactation ADFI and dietary energy and AA levels.

Sow lactation ADFI was divided into quintiles to analyze the relationship between lactation ADFI and sow and litter performance. Because multiparous sows had greater lactation ADFI and a wider feed intake range compared to gilts, they were analyzed separately using different intake groups. Separating into the quintile groups resulted in the following ADFI groups for gilts: < 5.30, 5.30 to 5.90, 5.90 to 6.40, 6.40 to 6.95, and * ≥ *6.95 kg/d. For multiparous sows, the ADFI groups were: < 6.00, 6.00 to 6.85, 6.85 to 7.45, 7.45 to 8.30, and* ≥ *8.30 kg/d.

Equations were developed to predict litter ADG and sow BW change from farrowing to weaning. Body weight at farrowing is the empty sow BW after farrowing. The predictor variables evaluated in the model to estimate litter ADG and sow BW change included ADFI, weaning age, litter size at d 2, litter size at weaning, NE intake (kcal/kg), dietary SID Lys, %, Met:Lys, Trp:Lys, Thr:Lys, Val:Lys, and Lys:CP. Histidine:Lys, Ile:Lys, and Leu:Lys ratio were not included as predictor variables in the model due to high correlation (*r* > 0.65) with SID Lys. For the estimate of litter ADG, sow BW change from farrow to weaning was used as a predictor variable, and for the estimate of sow BW change from farrowing to weaning, litter ADG was used as a predictor variable.

As an additional analysis, sow BW change over lactation was divided into three separate categories for gilts and sows with an equal number of animals in each category to compare changes in litter ADG at different levels of feed intake and sow lactation BW change. Gilt BW change categories were: ≤ −11.0, −11.0 to 1.4, and ≥ 1.4 kg/d. Multiparous sow BW change categories were: ≤ −3.3 kg, −3.33 to 13.3 kg, and ≥ 13.3 kg. The relationship between lactation ADFI, litter ADG, and sow BW change was graphed, with separate linear regression lines for each sow BW change category.

### NRC comparison

Predicted sow BW change and litter ADG from the regression equations were compared with values predicted from the NRC (2012) models. Inputs for both the regression equations and the NRC (2012) model were derived from average sow and litter performance in the dataset at increasing ADFI. The NRC (2012) lactating sow Excel model was used to predict BW change over lactation. To predict litter ADG, the NRC (2012) Equations 8–70 was used. The equation was algebraically rearranged to solve for litter ADG:


Litter ADG (g/d)=mean milk energy output (kcal/d)+90×litter size ÷ 4.92

Mean milk energy output was estimated for each sow in the dataset using NRC (2012) Equations 8–70. Then, the linear relationship between mean milk energy output and ADFI was determined:


Mean milk energy output, kcal/kg=548.19 ADFI, kg+9187.4

This relationship was used to predict mean milk energy output at increasing levels of lactation ADFI for the prediction of litter ADG.

### Statistical analysis

Performance data were analyzed by gilt and sow ADFI group using the lmer function of R software (version 1.4.171, R Foundation for Statistical Computing, Vienna, Austria). Lactation ADFI group was included in the model as a fixed effect. Trial was included in the model as a random effect, and weaning age was included as a covariate. Linear and quadratic orthogonal polynomial contrasts were used to evaluate the effect of increasing feed intake group. Discrete data were analyzed using a negative binomial distribution. Results are considered significant at *P* ≤ 0.05 and marginally significant at 0.05 < *P* ≤ 0.10.

The prediction equations were developed in R (Version 4.4.3) using the lmer function. To reduce multicollinearity among predictor variables, a correlation analysis was conducted using the corr function in R studio. When two variables had a correlation coefficient of greater than 0.65, one was excluded from the analysis based on model performance. The Kennard-Stone algorithm (Kennard and Stone 1969) was used to divide the database into a calibration (80%) and validation (20%) dataset. Backward stepwise regression was used to develop prediction equations for litter ADG and sow BW change over lactation. Trial was used as a random effect in the model. Model development began with linear terms, followed by the addition of linear and quadratic terms, and then expanded to include linear, quadratic, and all possible interaction terms. Terms were included in the model if they were statistically significant (*P* < 0.05). The model with the lowest Bayesian Information Criterion (BIC) was selected as the best fitting model. The coefficient of determination (*R*²) was calculated to determine the proportion of variation explained by the model, while mean bias and standard error of prediction (SEP) were used to assess model accuracy and repeatability, respectively, using the validation dataset.

## Results and discussion

### Lactation ADFI effect on litter ADG and sow BW change

In gilts, entry and post-farrowing BW varied by ADFI group, where BW initially decreased and then increased with greater ADFI, (quadratic; *P* < 0.05; Table 3) with gilts in the lowest and highest ADFI categories having similar BW. For gilts, entry and post-farrowing BW were not closely associated with lactation ADFI. Body weight at weaning increased (quadratic; *P* < 0.05; Table 3) with increasing ADFI. Gilt BW at weaning increased steadily with greater ADFI, increasing by about 1% with each ADFI group until the highest feed intake group, where there was a 4% increase in BW between gilts consuming 6.40 to 6.95 kg and gilts consuming more than 6.95 kg/d. Body weight loss from entry to weaning and farrow to weaning decreased (quadratic; *P* < 0.05) as ADFI increased. Body weight loss decreased from −12.5 kg in the lowest feed intake group to 0 kg in the highest feed intake group from farrow to weaning. No differences in WSI due to ADFI were observed in gilts.

**Table 3:** Effects of lactation feed intake on litter ADG and BW change—gilts.^a^

	Lactation ADFI^b^, kg						*P =*	
	≤ 5.30	5.30–5.90	5.90–6.40	6.40–6.95	≥ 6.95	SEM	Linear	Quadratic
**No. of gilts**	195	206	194	197	199			
**Parity**	1.0	1.0	1.0	1.0	1.0			
**ADFI^b^, kg**	4.8	5.7	6.2	6.7	7.5	0.03	< 0.001	0.991
**Gilt BW^c^, kg**								
**Entry**	225.9	223.2	220.7	222.3	226.8	6.76	0.856	0.003
**Farrow**	205.1	202.3	200.0	201.6	206.4	6.47	0.676	0.001
**Weaning**	193.0	195.6	197.1	198.5	206.5	7.49	< 0.001	0.033
**Gilt BW^c^ change, kg**								
**Entry to weaning**	−33.4	−27.7	−23.6	−23.8	−20.5	2.87	< 0.001	< 0.001
**Farrow to weaning**	−12.5	−6.8	−2.9	−3.1	0.0	2.78	< 0.001	0.032
**Lys intake, g/d**	46.5	57.0	61.9	66.4	74.0	2.18	< 0.001	0.004
**Lys intake, g/kg of litter gain**	22.4	24.6	27.7	27.2	28.7	1.26	< 0.001	0.110
**NE intake, Mcal/d**	12.0	14.1	15.4	16.6	18.7	1.79	< 0.001	0.866
**WSI^d^, d**	4.3	4.8	4.3	4.4	4.4	0.35	0.674	0.668
**Litter size, n**								
**d 2**	14.0	14.5	14.5	14.6	14.4	0.43	0.311	0.150
**Weaning**	12.5	13.1	12.9	13.5	13.3	0.39	0.023	0.412
**Litter weight, kg**								
**d 2**	20.8	21.1	21.2	21.5	21.7	0.72	0.194	0.919
**Weaning**	69.7	70.9	70.1	74.1	75.8	3.62	< 0.001	0.801
**Mean pig BW^c^, kg**								
**d 2**	1.49	1.47	1.47	1.48	1.52	0.044	0.181	0.026
**Weaning**	5.35	5.43	5.47	5.51	5.70	0.144	< 0.001	0.236
**Litter ADG^e^, kg**	2.39	2.59	2.55	2.73	2.80	0.073	< 0.001	0.675
**Pig ADG^f^, g**	196	203	203	208	215	8.64	< 0.001	0.741

a A total of 991 gilts from eight studies were divided into quintiles of lactation ADFI, with each quintile representing 20% of the population, to evaluate the effects of feed intake on sow BW change during lactation and litter growth performance.

b ADFI, average daily feed intake.

c BW, body weight.

d WSI, wean to service interval.

e ADG, average daily gain.

In multiparous sows, BW at entry and farrowing varied among ADFI groups, where BW decreased through the intermediate ADFI group and then plateaued thereafter (quadratic; *P* < 0.05; Table 3). Multiparous sow BW at weaning decreased (quadratic; *P* < 0.05; Table 4) as lactation ADFI increased from less than 6.0 to 7.45 kg, then increased as ADFI increased from 7.45 to greater than 8.30 kg, with the highest ADFI group having the greatest BW at weaning. Body weight loss from entry to weaning decreased (quadratic; *P* < 0.05) as ADFI increased in multiparous sows, with the greatest reduction in BW loss occurring between sows consuming less than 6.0 kg and 6.00 to 6.85 kg. However, BW loss from farrowing to weaning decreased (linear, *P* < 0.05) with increasing ADFI group, with sows gaining BW when ADFI was greater than 6.85 kg. For multiparous sows, no differences in WSI were observed between sow ADFI group. When comparing BW change for gilts and multiparous sows, over 80% of gilts lost BW from farrow to weaning, while over half of the multiparous sows gained BW during lactation. Multiparous sows with an ADFI of greater than 6.85 kg gained BW during lactation. In contrast, gilts with the highest ADFI, greater than 6.95 kg, maintained BW over lactation.

**Table 4:** Effects of lactation feed intake effect on litter ADG and sow BW change—multiparous sows.^a^

	Lactation ADFI^b^, kg						*P =*	
	≤ 6.00	6.00–6.85	6.85–7.45	7.45–8.30	≥ 8.30	SEM	Linear	Quadratic
**No. of sows**	678	683	674	676	682			
**Parity**	3.6	3.5	3.5	3.5	3.8			
**ADFI^b^, kg**	5.3	6.5	7.1	7.8	9.1	0.04	< 0.001	0.947
**Sow BW^c^, kg**								
**Entry**	288.9	279.8	271.0	273.0	272.2	8.93	< 0.001	< 0.001
**Farrow**	261.6	253.8	245.3	247.1	245.6	8.43	< 0.001	< 0.001
**Weaning**	252.6	251.2	248.4	252.4	257.9	10.17	0.006	< 0.001
**Sow BW^c^ change, kg**								
**Entry to weaning**	−36.2	−28.5	−22.7	−20.7	−14.3	3.97	< 0.001	0.038
**Farrow to weaning**	−8.9	−2.5	2.9	5.1	12.1	4.00	< 0.001	0.601
**Lys intake, g/d**	52.1	65.3	71.4	78.2	90.7	2.17	< 0.001	0.450
**Lys intake, g/kg of litter gain**	22.0	26.5	27.7	29.9	33.1	1.50	< 0.001	0.149
**NE intake, Mcal/d**	13.3	16.3	17.8	19.6	22.5	2.22	< 0.001	0.440
**WSI^d^, d**	5.2	6.8	6.2	5.0	5.7	1.64	0.843	0.531
**Litter size, *n***								
**d 2**	14.5	14.5	14.4	14.5	14.5	0.28	0.905	0.818
**Weaning**	12.5	12.7	12.8	12.8	12.9	0.35	0.030	0.491
**Litter weight, kg**								
**d 2**	22.7	22.8	22.7	23.0	22.9	0.83	0.162	0.821
**Weaning**	74.8	76.9	78.6	79.9	81.5	2.11	< 0.001	0.461
**Mean pig BW^c^, kg**								
**d 2**	1.57	1.58	1.58	1.59	1.59	0.051	0.162	0.930
**Weaning**	5.97	6.06	6.16	6.22	6.33	0.127	< 0.001	0.902
**Litter ADG^e^, kg**	2.66	2.76	2.85	2.90	2.99	0.082	< 0.001	0.488
**Pig ADG^e^, g**	220	224	230	232	238	7.5	< 0.001	0.732

a A total of 3393 sows from eight studies were divided into quintiles of lactation ADFI, with each quintile representing 20% of the population, to evaluate the effects of feed intake on sow BW change during lactation and litter growth performance.

b ADFI, average daily feed intake.

c BW, body weight.

d WSI, wean to service interval.

e ADG, average daily gain.

Several other studies have observed a positive relationship between lactation ADFI and maternal BW change over lactation (Eissen et al. 2003; Strathe et al. 2017; Estrada et al. 2024). Increasing lactation feed intake in gilts is important to increase litter weight gain, and reduce BW loss, resulting in improved subsequent reproductive performance. Decreasing BW loss during the first lactation also increases litter size during the next reproductive cycle (Eissen et al. 2003). Farmer et al. (2016) also noted that proper gilt body condition is important for mammogenesis because gilts that are too thin or obese have decreased mammary development compared to gilts in proper body condition, impacting lifetime milk production.

As expected, for both gilts and multiparous sows, Lys and NE intake and Lys intake per kg of litter gain increased (linear; *P* < 0.05) as ADFI increased. The lower Lys intake per kg of litter gain at low ADFI suggests that gilts and sows with low feed and Lys intake are more efficient at utilizing dietary Lys for litter gain. Feeding sows a low crude protein diet in lactation has been shown to increase N and AA retention and AA utilization efficiency (Huber et al. 2015). Similarly, restricted fed sows given 4 kg per day had greater lactation efficiency, producing more milk per kg of feed intake, than sows given 8 kg per day (De Bettio et al. 2016). Bergsma et al. (2009) observed that sows with lower feed intake were more efficient at utilizing energy toward milk production and litter gain compared to sows with higher feed intake, similar to the results of the current analysis. However, even though gilts and sows with lower lactation feed intake appeared more efficient, they had lower litter growth performance and mobilized more body reserves than gilts and sows with higher feed intake. The Lys intake per kg of litter gain value only accounts for dietary Lys, it does not account for Lys that is mobilized from maternal muscle tissue and used for milk production, making it difficult to determine whether sows were more efficient at lower lactation feed intakes.

In gilts and multiparous sows, no differences in litter size or litter weight at d 2 were observed among lactation ADFI groups. However, in gilts on d 2, pig BW was slightly lower for sows in the middle ADFI categories than sows with the highest ADFI (quadratic; *P* < 0.05). This difference is likely due to chance, because d 2 pig weights were recorded prior to lactation feed intake. In contrast, no differences in pig weight at d 2 were observed between ADFI groups for multiparous sows. For both gilts and multiparous sows, weaning litter size, weaning litter and pig weight, as well as litter and pig ADG increased (linear; *P* < 0.05) with increasing ADFI. The greatest increase in litter ADG occurred as ADFI increased from the lowest to the second-lowest intake group in both gilts and multiparous sows. Similar to the results of the current analysis, Koketsu et al. (1996b) observed an increase in litter size at weaning as lactation ADFI increased. Increased litter size and gain are associated with increased feed intake during lactation (Eissen et al. 2000).

Body condition at farrowing can also affect lactation feed intake. This analysis indicates that multiparous sows that enter farrowing with the highest BW consume less feed, have greater BW loss over lactation, and lower litter growth performance, which has been observed in previous trials (Revell et al. 1998; Cools et al. 2014). When comparing litter growth performance in the present analysis, multiparous sows had greater pig and litter ADG at similar ADFI, and overall higher litter growth performance compared to gilts.

The analysis of lactation ADFI for gilts and multiparous sows indicates that as ADFI, and therefore NE and Lys intake, increases, sow BW loss over lactation decreases and litter ADG increases. Similar results have been observed in the literature (De Bettio et al. 2016; Choi et al. 2017; Estrada et al. 2024), with increased feed intake improving litter growth performance and minimizing sow BW loss. De Bettio et al. (2016) observed that restricted fed sows consuming 4.14 kg/d had greater BW loss (28.2 vs. 7.8 kg) and lower litter ADG (2.43 vs. 2.70 kg) compared to ad libitum fed sows consuming 6.43 kg/d; however, no differences in WSI were observed, which was also observed in the current analysis. Similar to the results of the current analysis, Estrada et al. (2024) observed an increase in weaning weight and a decrease in sow BW loss during lactation when classifying sows by low (5.48 kg/d), average (6.85 kg/d), and high (8.17 kg/d) ADFI. Choi et al. (2017) reported that when sows were fed diets formulated with higher energy and amino acid concentrations compared to NRC requirement estimates, sow BW loss decreased (19.6 vs. 23.9 kg) and pig ADG increased (229 vs. 203 g/d). These improvements occurred despite similar ADFI, indicating that nutrient density, rather than feed intake alone, influences sow and litter performance. Sow lactation diets are formulated to meet the requirements of the average sow in the herd. Sows that have lower feed intake than the herd average will have deficient amino acid and energy intake, while sows with high feed intake may be exceeding their requirements resulting in excess nutrient excretion. This is especially important in gilts because they have approximately 15% lower ADFI compared to multiparous sows (Strathe et al. 2017).

In response to inadequate nutrient intake, sows mobilize body reserves to support lactation demands. However, the ability of sows to compensate for insufficient nutrient intake by mobilizing body reserves has limits, as excessively low feed intake reduces milk production and litter growth rate even when body reserves are mobilized (Theil et al. 2023) compared to sows with higher feed intake. Theil et al. (2023) observed that when sows lose more than 10% of BW during lactation and energy is deficient by more than 3.6 Mcal/d, milk production is reduced. Based on the results of the current study, NE required for maintenance and milk production were estimated using equations from Noblet and Etienne (1989) and Li et al. (2024). Estimated NE required for maintenance and milk production per d were compared with average NE intake per d. In gilts with the lowest ADFI, NE intake averaged 5.7 Mcal/d below the estimated requirement for maintenance and milk production, leading to BW loss during lactation. In contrast, sows with the highest ADFI consumed 0.8 Mcal/d more than their estimated requirement for maintenance and lactation, resulting in BW gain during lactation.

### Regression analysis

#### Gilt and sow BW change over lactation

Multivariable regression analysis was used to predict litter ADG and gilt and sow BW change over lactation (Table 5). For equations to predict both litter ADG and maternal BW change, the simple linear model had the best fit, as determined by the lowest BIC. The stepwise models to predict maternal BW change and litter ADG were validated using 135 datapoints for gilts and 484 datapoints for sows, and results indicated a positive relationship between observed and predicted values, with points following the line of best fit (Figs 1–4). The variables included in the regression model to predict sow BW change over lactation explain 40% and 36% of the variation for gilts and multiparous sows, respectively.

**Figure 1:**
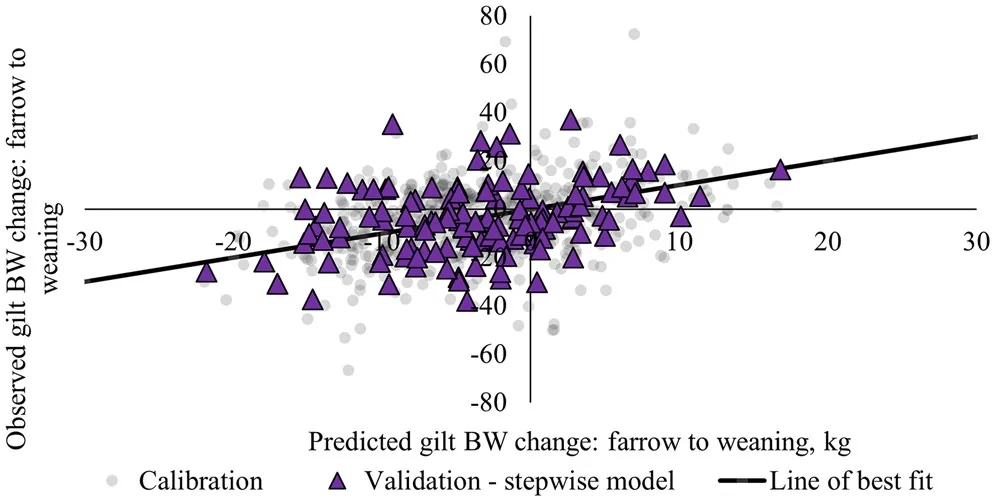
Regression analysis of the relationship between predicted and observed gilt BW weight change, based on the prediction model selected through stepwise selection. Gray circles represent data points used for model calibration (*n* = 531) and purple triangles represent points used for model validation (*n* = 135). The solid line represents the line of best fit (predicted = observed).

**Figure 2:**
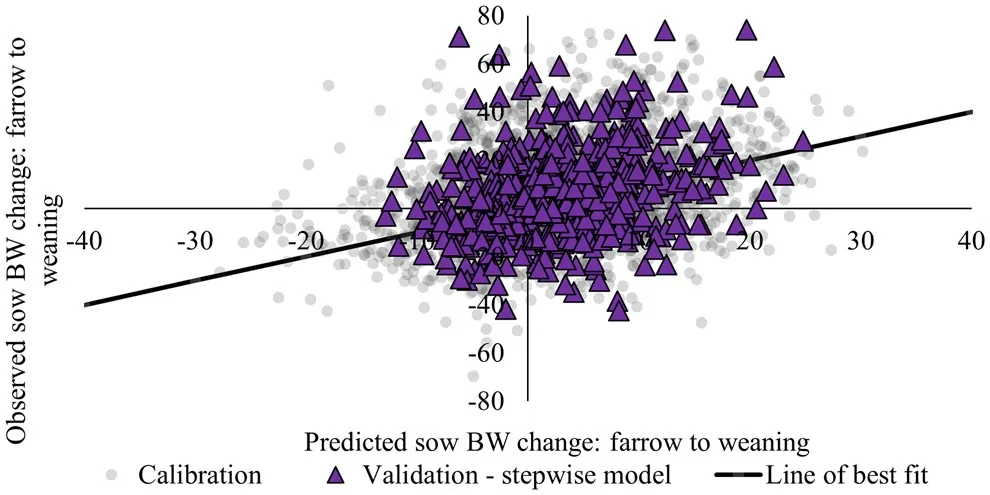
Regression analysis of the relationship between predicted and observed sow BW weight change, based on the prediction model selected through stepwise selection. Gray circles represent data points used for model calibration (*n* = 1935) and purple triangles represent points used for model validation (*n* = 484). The solid line represents the line of best fit (predicted = observed).

**Figure 3:**
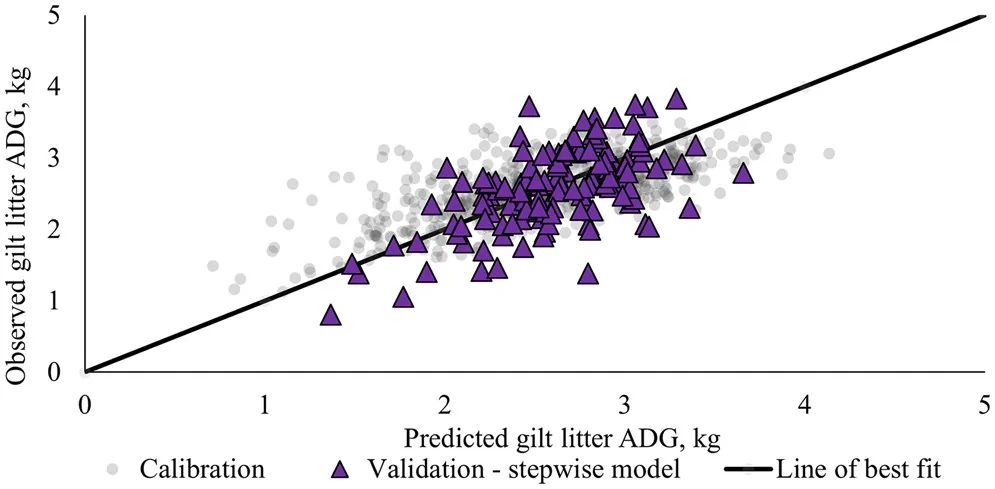
Regression analysis of the relationship between predicted and observed gilt litter ADG, based on the prediction model selected through stepwise selection. Gray circles represent data points used for model calibration (*n* = 531) and purple triangles represent points used for model validation (*n* = 135). The solid line represents the line of best fit (predicted = observed).

**Figure 4:**
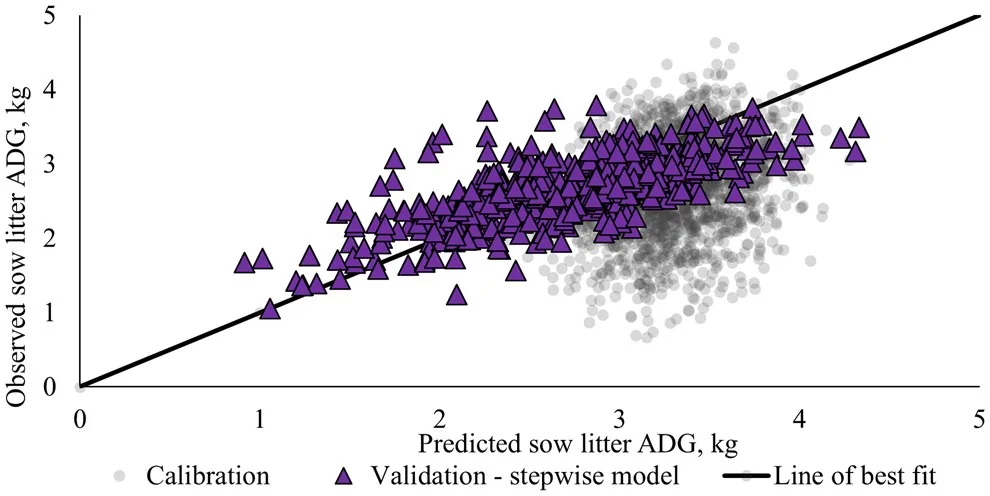
Regression analysis of the relationship between predicted and observed sow litter ADG, based on the prediction model selected through stepwise selection. Gray circles represent data points used for model calibration (*n* = 1935) and purple triangles represent points used for model validation (*n* = 484). The solid line represents the line of best fit (predicted = observed).

**Table 5:** Regression models to predict litter ADG and sow BW change from farrow to weaning in gilts and multiparous sows.

Variable^a^	Equation	*R*2
**Sow BW change farrow to weaning, kg- gilts**	3.580 + (4.738 × ADFI, kg)—(9.734 × Litter ADG, kg)—(0.569 × Wean age)	0.40
**Sow BW change farrow to weaning, kg- multiparous sows**	−30.936 + (5.188 × ADFI, kg) – (8.316 × Litter ADG, kg) + (1.404 × Pig count d 2)	0.36
**Litter ADG, kg- gilts^b^^,^^c^**	0.042 + (0.107 × ADFI, kg) + (0.550 × SID Lys) − (0.009 × Gilt BW Change farrow to weaning, kg) − (0.122 × Pig count d 2) + (0.236 × Pig count wean)	0.58
**Litter ADG, kg- multiparous sows**	0.288 + (0.060 × ADFI, kg) − (0.004 × Sow BW change farrow to weaning, kg) − (0.118 × Pig count d 2) + (0.280 × Pig count weaning) + (0.013 × Weaning age)	0.59

a ADG, average daily gain; ADFI, average daily feed intake; SID, standardized ileal digestibility.

b Dietary SID Lys should be expressed as a percentage.

c Pig count d 2 = count of pigs in a litter at 24 h postpartum, after cross fostering.

The first variable selected through backward selection to predict both gilt and sow BW change over lactation was ADFI. If all other variables are held constant, when ADFI increases by 1 kg, BW loss over lactation decreases by 4.74 and 5.19 kg for gilts and multiparous sows, respectively. Litter ADG was included in the model as a significant predictor for both the gilt and multiparous sow equations. For both equations, as litter ADG increases, sow BW loss increases over lactation. For every 100 g increase in litter ADG, BW loss over lactation increases by 0.973 and 0.832 kg for gilts and multiparous sows, respectively, when all other variables are held constant. Average daily feed intake and litter ADG have the greatest impact on sow BW change over lactation. Together, lactation ADFI and litter ADG accounted for the majority of the variation in sow BW change, explaining 21.8% in gilts and 18.1% in multiparous sows, respectively. For both gilts and multiparous sows, increased ADFI reduces sow BW loss, indicating that increased nutrient intake decreases maternal body reserve mobilization during lactation. Generally, sows lose BW during lactation because of high milk yield and inadequate nutrient intake. The slope for ADFI was greater in older parity sows compared to gilts, suggesting that multiparous sows exhibit greater sensitivity to increases in feed intake, allocating a larger proportion toward maternal body gain or reduced BW loss. The regression equation indicates that for both gilts and multiparous sows, an increase in litter ADG increases sow BW loss over lactation due to partitioning of nutrients toward milk production and mobilization of body reserves.

Weaning age was a significant predictor for gilt BW change, with increased weaning age increasing gilt BW loss. Pig count at d 2 was a significant predictor for multiparous sows, with increased pig count at d 2, decreasing sow BW loss over lactation. Weaning age alone explains only 1.0% of the variation in BW change for gilts, and pig count at d 2 explains only 1.2% of the variation for multiparous sows.

Similar to equations developed by Strathe et al. (2017) and Holen et al. (2022), ADFI was included as a significant predictor of gilt and multiparous sow BW. Litter size after equalization was included in the equation developed herein for multiparous sows, and equations developed by Strathe et al. (2017) and Holen et al. (2022) but not for the prediction of gilt BW change. Backfat loss over lactation was included as a significant predictor in equations developed by Strathe et al. (2017) but was not recorded in all studies used to develop the equations in the current analysis. The addition of gilt and sow lactation backfat loss could potentially improve the *R*^2^ of the current regression equation.

#### Litter ADG

The variables included in the regression model to predict litter ADG explained 58% and 59% of the variation in litter ADG for gilts and multiparous sows, respectively (Table 5). For equations developed to predict litter ADG, the first variable included through backward selection was ADFI. The regression equation indicates that a 1 kg increase in ADFI leads to a greater increase in litter ADG in gilts vs. multiparous sows (107 vs. 60 g). Lactation ADFI was a poor single predictor of litter ADG, explaining only 6.0% of the variation in litter ADG for gilts and 3.9% in multiparous sows. The regression equations indicate that as sow BW loss over lactation increases by 1 kg, litter ADG increases by 9 and 4 g, respectively, when all other variables are held constant. Sow BW change over lactation alone explains only 8.8% and 3.3% of the variation in litter ADG for gilts and multiparous sows, respectively. Pig count at d 2 and pig count at weaning were included in the model as significant predictors of litter ADG, with an increase in pig count at d 2 reducing litter ADG in both prediction equations and an increase in pig count at weaning increasing litter ADG. Across both parity groups, pig count at weaning was the strongest single predictor of litter ADG, explaining 36.2% of the variation in gilts and 39.5% in multiparous sows, while pig count at day 2 explained 13.0% and 12.3%, respectively. Formulated SID Lys percent was included as a significant predictor for litter ADG for gilts only. This is likely because gilts have a higher SID Lys requirement estimate for maternal body protein compared to multiparous sows to support maternal growth (Trottier et al. 2014). As dietary SID Lys level increases by 0.1%, litter ADG increases by 55 g in gilts. However, dietary SID Lys level only accounted for 1.1% of the variation in litter ADG in this dataset. Through backward selection, weaning age was selected as the last significant factor to predict litter ADG of multiparous sows, with an increase in weaning age increasing litter ADG. As weaning age increases by 1 d, litter ADG increases by 13 g, but accounts for only 0.5% of the variation in litter ADG.

Other regression equations developed to predict litter ADG also have found that litter ADG increases as ADFI and litter size at weaning increase (Strathe et al. 2017; Holen et al. 2022). In contrast to the regression equations developed herein, equations developed to predict litter ADG by Holen et al. (2022) included dietary net energy as a predictor of litter ADG; however, net energy was not a significant predictor in the current model. Equations developed by Strathe et al. (2017) included backfat loss and ADFI over specific days of lactation.

The regression equation developed herein indicates that a one kg increase in ADFI increases litter ADG at a greater rate in gilts compared to multiparous sows (107 vs. 60 g); although the difference is relatively small. This is a surprising observation because gilts are still growing and partitioning nutrient intake between milk production and maternal growth. Pluske et al. (1998) observed that gilts will use nutrients from increased feed intake to improve body condition rather than increase milk production. Cozannet et al. (2018) determined that gilts prioritize increased energy intake during lactation for their own body growth, whereas multiparous sows use it to support higher milk production and litter growth, likely because gilts are still maturing during their first lactation. A study evaluating the effects of energy intake of gilts in lactation found that as energy intake increased, no differences in litter weight at weaning were observed, suggesting similar milk production, but maternal BW gain over lactation increased as energy intake increased, again highlighting that gilts prioritize maternal BW gain over milk production (Pluske et al. 1998). Equations developed by Strathe et al. (2017) indicate that increases in ADFI result in greater improvements in litter ADG for multiparous sows compared to gilts. Gilt feed intake is limited by physical capacity to consume feed (Lewis and Bunter 2011), and they are less efficient at converting nutrients into milk compared to older parity sows (Pluske et al. 1998). Gilts have a smaller udder compared to sows and are still undergoing mammary gland development during the first lactation (Balzani et al. 2016), leading to lower milk yield (Rempel et al. 2023).

A potential explanation for the differing ADFI coefficients between gilts and multiparous sows in predicting litter ADG in the analysis herein is that gilts typically consume less feed during lactation. Their lower nutrient intake constrains litter growth, causing increases in ADFI to yield greater marginal improvements in litter ADG compared with older sows. Another potential explanation is that through genetic selection, gilts are more efficient at utilizing nutrients and can support larger litters. When comparing the results of Pluske et al. (1998) to the current analysis, the average gilt BW at farrowing was 177 kg, compared to 203 kg in the current analysis. Heavier gilts at farrowing have greater body reserves that can be mobilized to support lactation. Likewise, litter size at weaning increased from 8.6 (Pluske et al. 1998) and 10.2 (Cozannet et al. 2018) pigs in previous studies to 13.1 pigs in the current analysis. These differences in gilt and litter characteristics could play a role in the differences in responses observed in the current analysis for nutrient portioning of gilts.

Predicted maternal BW change and litter ADG between the equations developed for gilts and multiparous sows developed in the analysis herein and NRC (2012) prediction models were compared (Figs 5 and 6). Inputs for the equations were derived from the analyzed data and their observed relationships as ADFI increased (Tables 6 and 7). The NRC (2012) sow lactation BW change model predicted substantially greater BW loss for both gilts and multiparous sows at low lactation ADFI compared with the equations developed in the current analysis, −17.7 vs. −4.0 and −27.5 vs. −3.1 at an ADFI of 5.5 kg for gilts and multiparous sows, respectively. The developed prediction equation indicates that modern sows are more efficient and lose less BW over lactation at low feed intake. When comparing the regression results for gilts and multiparous sows, gilt and multiparous sow BW change is similar, with multiparous sows losing slightly less BW at low feed intake levels below 6.0 kg. As ADFI increased, multiparous sows lost less BW and began to gain BW at approximately 6.5 kg ADFI, whereas gilts began gaining BW at around 7.5 kg ADFI. As litter ADG increases, there is a greater change in maternal BW for gilts compared to sows.

**Figure 5:**
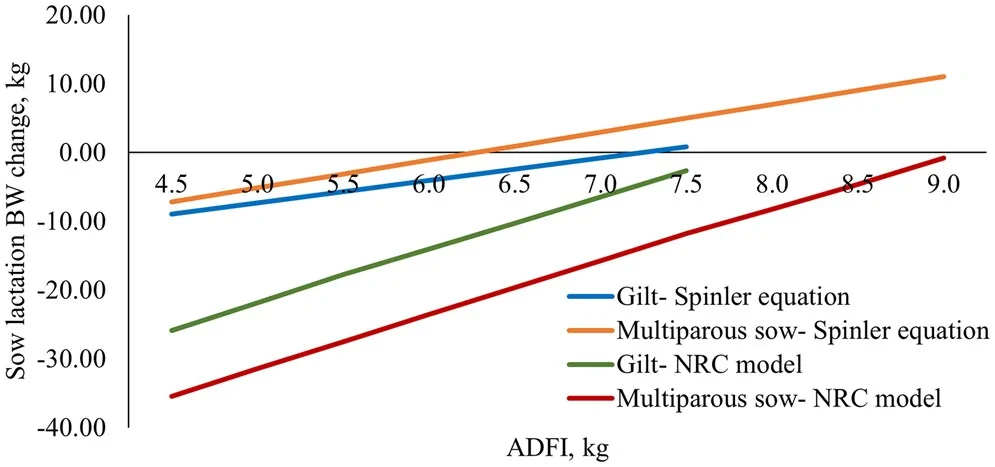
Comparison of predicted sow body weight (BW) change during lactation using equations developed in the present analysis (Gilts = 3.580 + (4.738 × ADFI, kg) – (9.734 × Litter ADG, kg) – (0.569 × Wean Age); Multiparous sows = −30.936 + (5.188 × ADFI, kg) – (8.316 × Litter ADG, kg) + (1.404 × Pig Count d 2)) vs. NRC (2012) excel based model predictions for gilts and multiparous sows. Table 5 outlines the inputs to the regression equations.

**Figure 6:**
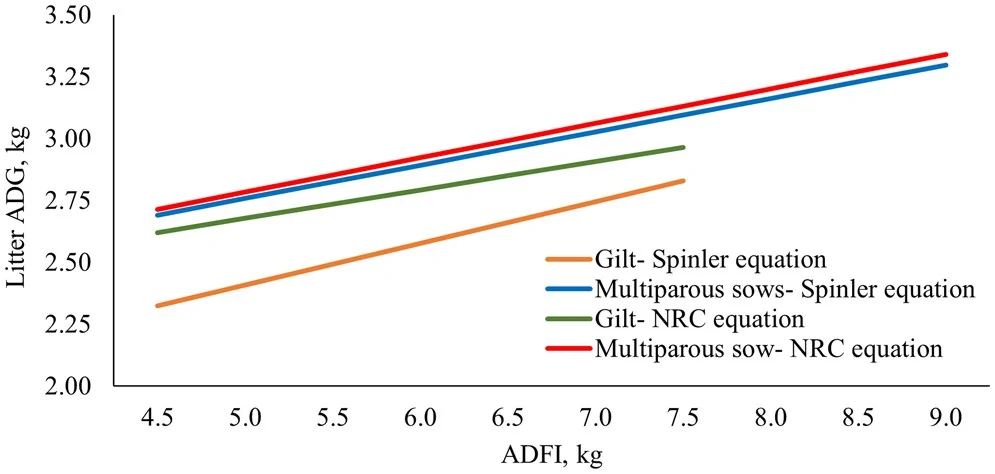
Comparison of liter average daily gain (ADG) using equations developed in the present analysis (Gilts = 0.042 + (0.107 × ADFI, kg) + (0.550 × SID Lys) − (0.009 × Gilt BW change farrow to weaning, kg) − (0.122 × Pig Count d 2) + (0.236 × Pig Count Wean); Multiparous sows = 0.288 + (0.060 × ADFI, kg) − (0.004 × Sow BW change farrow to weaning, kg) − (0.118 × Pig Count d 2) + (0.280 × Pig Count Wean) + (0.013 × Wean Age)) vs. NRC (2012) prediction Equation 8–70 for gilts and multiparous sows. Table 6 outlines the inputs to the regression equations.

**Table 6:** Inputs used to predict lactation sow body weight change across increasing sow lactation average daily feed intake using equations developed in the current analysis (Spinler) and NRC (2012) model outputs.^a^

	ADFI, kg			
Item	4.5	6.0	7.5	9.0
**Spinler equation- Gilts^b^**				
**Litter ADG, kg**	2.34	2.52	2.70	–
**Weaning age, d**	19.4	20.2	21.1	–
**Spinler equation- Multiparous sows^c^**				
**Litter ADG, kg**	–	2.60	2.81	3.01
**Pig Count d 2**	–	14.51	14.51	14.51
**NRC model- Gilts^d^**				
**Sow BW^e^ farrow, kg**	200.5	199.8	199.1	–
**Pig ADG, g/d**	206	211	216	–
**Average litter size^f^**	13.34	13.56	13.79	–
**NRC model- Multiparous sows^d^**				
**Sow BW^e^ farrow, kg**	–	258.2	250.8	243.4
**Pig ADG, g/d**	–	229	235	241
**Average litter size^f^**	–	13.74	13.97	14.20

a Comparison of prediction equation found in Fig. 5.

b = 3.580 + (4.738 × ADFI, kg) – (9.734 × Litter ADG, kg) – (0.569 × Wean age).

c = −30.936 + (5.188 × ADFI, kg) – (8.316 × Litter ADG, kg) + (1.404 × Pig count d 2).

d ME of diet, kg/kcal = 3292.

e BW, body weight.

f Average litter size from litter d 2, after litter equalization, to weaning.

**Table 7:** Inputs used to predict litter average daily gain (ADG) across increasing sow lactation average daily feed intake (ADFI) using equations developed in the current analysis (Spinler) and NRC (2012) model outputs.^a^

	ADFI, kg			
Item	4.5	6.0	7.5	9.0
**Spinler equation- Gilts^b^**				
**SID Lys, %**	1.02	1.02	1.02	–
**Gilt BW^c^ change, kg^d^**	−10.2	−5.6	−0.9	–
**Pig count d 2**	14.95	14.95	14.95	–
**Pig count weaning**	12.59	13.16	13.54	–
**Spinler equation- Multiparous sows^e^**				
**Sow BW^c^ change, kg^d^**	–	−2.6	5.5	11.2
**Pig count d 2**	–	14.51	14.51	14.51
**Pig count weaning**	–	12.98	13.43	13.88
**Weaning age, d**	–	20	20	20
**NRC Eq. 8–70- Gilts**				
**Milk energy output, kcal/d**	11,654	12,477	13,299	–
**Average litter size^f^**	13.77	14.06	14.34	–
**NRC Eq. 8–70- Multiparous sows**				
**Milk energy output, kcal/d**	–	12,803	13,809	14,816
**Average litter size**	–	13.74	13.97	14.20

a Comparison of prediction equations found in Fig. 6.

b = 0.042 + (0.107 × ADFI, kg) + (0.550 × SID Lys) − (0.009 × Gilt BW change farrow to weaning, kg) − (0.122 × Pig count d 2) + (0.236 × Pig count wean).

c BW, body weight.

d Maternal BW change from farrow to weaning.

e = 0.288 + (0.060 × ADFI, kg) − (0.004 × Sow BW change farrow to weaning, kg) − (0.118 × Pig count d 2) + (0.280 × Pig count wean) + (0.013 × Wean age).

f Average litter size from litter d 2, after litter equalization, to weaning.

Equation 8–70 from the 2012 NRC was used to predict litter ADG and compared to the equation developed in the current analysis. The NRC equation predicted a higher litter ADG compared to the equation developed in this analysis for gilts. Predicted values were much closer for multiparous sows, only a 0.02 kg difference for multiparous sows compared to 0.21 kg for gilts with an ADFI of 6.0 kg. The higher predicted litter ADG for gilts using the NRC (2012) equation compared to the equation developed in the current analysis was a surprising result because modern gilts have higher milk production and higher litter gain. The NRC prediction equation was developed using an average litter size smaller than that observed in the current analysis. When looking at the data used to develop the NRC equation, litters were equalized to 9 or 10 pigs. In contrast, the average litter size at equalization in the current analysis was 14.5. This likely contributes to the equation overestimating litter ADG for gilts, as the litter sizes in this study fall outside the range used to develop the NRC model. Koketsu et al. (1996a) observed that sows with an ADFI of 5.4 kg during lactation weaned litters averaging 53.3 kg and 8.7 pigs. In comparison, sows in the lowest ADFI group, 5.3 kg ADFI, weaned heavier litters averaging 76.8 kg and 12.5 pigs. This comparison highlights the substantial improvement in sow production over time, as modern sows are capable of producing more milk and raising larger litters at similar feed intake.

The comparison of predicted litter ADG of gilts and sows for the developed regression equation in this analysis indicates that sows had a greater litter ADG compared to gilts. However, the slope of the line for multiparous sows is flatter than that for gilts as ADFI increases due to multiple factors. In the regression equation, litter ADG is primarily influenced by the number of pigs weaned, and gilts in this dataset had larger litter sizes at weaning compared to sows. Lactation ADFI is also a key factor, with the equation indicating that litter ADG increases more rapidly with rising ADFI in gilts than in sows. Additionally, sows exhibit greater BW gain at high ADFI levels, which in turn is associated with lower litter ADG due to increased nutrient partitioning toward maternal gain rather than milk production.

### Relationship between BW gain and BW loss on litter ADG

The analysis indicates that ADFI affects both litter ADG and sow BW change over lactation. However, the analysis also indicates a complex interaction among the three variables, such that the effect of ADFI on litter ADG cannot be interpreted without also considering sow BW change, and vice versa. Figures 7 and 8 illustrate the relationship between lactation ADFI, litter ADG, and sow BW change over lactation for gilts and multiparous sows, respectively. Maternal BW change over lactation was divided into 3 categories, with an equal number of animals in each category, for gilts and multiparous sows and the relationship between ADFI and litter ADG was compared within BW category. The figures illustrate that even at similar ADFI, sows can lose, maintain, or gain BW during lactation. Gilts and sows that lose BW over lactation and have high feed intake have the highest litter ADG. Strathe et al. (2017) observed that sows with high milk production had both high feed intake and high body reserve mobilization, suggesting that their physical capacity to consume feed may limit overall performance. Hooyberghs et al. (2025) observed that sows that lose BW over lactation have a higher piglet survival rate, likely due to increased milk production. The difference between nutrient intake and nutrients required for maintenance and milk production determines the sows demand to mobilize body reserves for milk production (Theil et al. 2023). Closer examination of the data in the present analysis showed that gilts and sows with similar feed intake that lost BW during lactation had greater litter ADG and weaned larger litters compared with those that gained BW. When sows lose body weight during lactation, it indicates that body reserves are being mobilized to use toward milk synthesis or for maintenance requirements. When sows have both high nutrient intake and mobilize body reserves, they have more nutrients available for milk synthesis, likely increasing milk yield.

**Figure 7:**
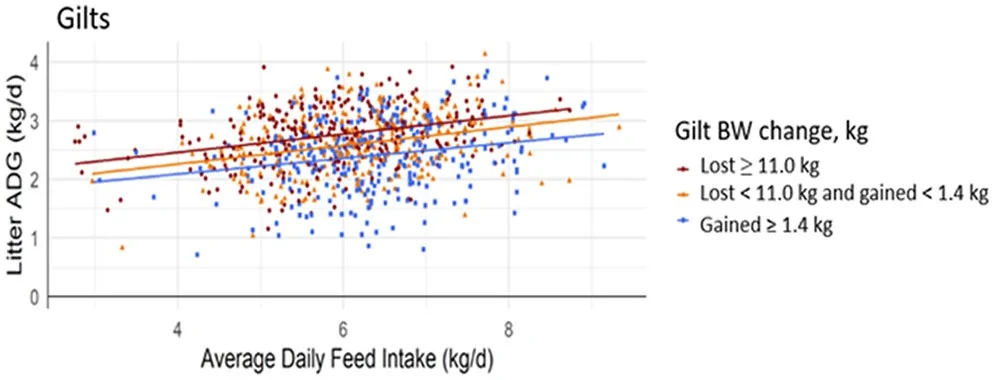
Relationship between average daily feed intake (ADFI; kg/d), litter average daily gain (ADG; kg/d), and body weight (BW) change in gilts. Gilts were divided into three categories based on body weight change from farrow to weaning (BW change, kg): ≤ −11.0 kg (dark red), −11.0 to 1.4 kg (orange), and ≥ 1.4 kg (blue). Points represent individual gilts, and the lines show linear regression trends for each BW change group.

**Figure 8:**
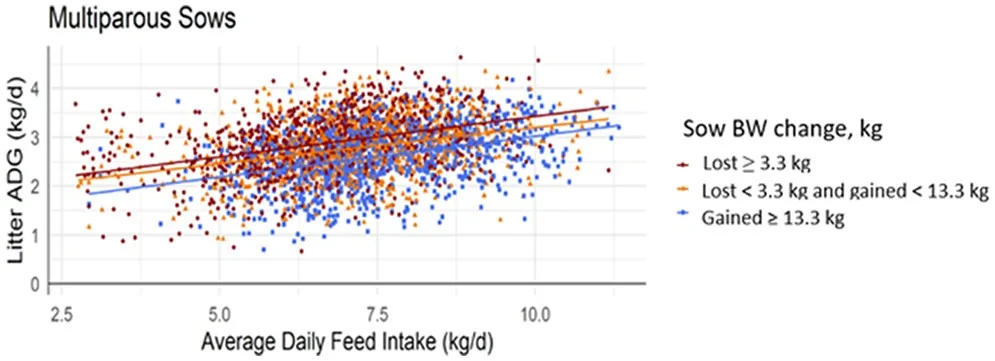
Relationship between average daily feed intake (ADFI; kg/d), litter average daily gain (ADG; kg/d), and body weight (BW) change in multiparous sows. Sows were divided into three categories based on body weight change from farrow to weaning (BW change, kg): ≤ −3.3  kg (dark red), −3.3 to 13.3 kg (orange), and ≥ 13.3 kg (blue). Points represent individual sows, and the lines show linear regression trends for each BW change group.

In the current analysis, gilts and sows that lost BW over lactation were heavier at entry and had a greater litter weight at d 2 and weaning compared to sows that had moderate BW change or gained BW over lactation. However, litter size at d 2 was similar between sows that lost and gained BW over lactation. A potential explanation for why sows lose BW over lactation compared to sows that gain BW with similar ADFI is that sows that lose BW had a heavier litter and average pig weight at d 2, increasing nursing pressure. Sows raising average birth weight pigs had greater BW loss over lactation compared to sows raising light birth weight pigs, even when feed intake was similar (Hawe et al. 2020). Larger piglets are more effective at stimulating milk production and emptying milk from teats (Grahofer and Plush 2023), leading to increased total milk yield.

Another possible explanation is that sows that lost BW during lactation were heavier at entry and farrow compared to sows that gained BW at similar ADFI levels. A study comparing performance of restricted vs. ad libitum fed sows, observed that milk production and body reserve mobilization was not dependent solely on lactation feed intake (Costermans et al. 2020). The results of Costermans et al. (2020) and the current analysis indicate that feed intake is important, but body reserves at farrowing are an independent driver of milk production. A sow that is heavier at farrowing has more body reserves to mobilize to increase milk production compared to leaner sows with similar feed intake. Thongkhuy et al. (2020) observed that sows with greater than 15 mm of backfat at entry to farrowing had greater backfat loss during lactation and higher milk yield from d 3 to 10 of lactation than sows with less backfat at entry.

While increased body reserves at farrowing can support greater milk production, excessive body fat can have negative consequences for sow health and reproductive outcomes. Sows that are overweight have an increased incidence of stillborns (Gourley et al. 2020) and lameness (Knage-Rasmussen et al. 2014). A quadratic increase in litter gain was observed as backfat increased from less than 10 mm to 22 mm, but decreased when backfat exceeded 22 mm for gilts and sows due to decreased feed intake (Kim et al. 2015). Clowes et al. (2003) observed that sows that lost greater than 10% of their BW during lactation had lower milk production and litter gain compared to sows that lost less than 10% of their BW during lactation. In the analysis herein, gilts and sows in the highest BW loss categories lost 9.6% and 5.6% of their BW, respectively, which likely contributes to the lack of a negative response in litter performance in the highest BW loss category. In the current analysis, only 17% of gilts and 5% of sows lost greater than 10% of their BW during lactation.

## Conclusion

In conclusion, these findings highlight that lactation ADFI, litter ADG, and sow BW change are intricately connected, emphasizing the importance of considering them together to fully understand sow and litter performance. For both gilts and multiparous sows, greater ADFI was associated with reduced body weight loss and improved litter performance. However, over half of the multiparous sows included in the analysis gained BW from farrow to weaning and 80% of gilts lost BW. The regression equation shows that high feed intake reduces BW loss; however, gilts experience greater BW loss when supporting litters with high ADG compared to older parity sows. Litter ADG increases as ADFI and litter size increase, with gilt litters having a greater improvement in growth compared to multiparous litters as ADFI increases. Sow BW change during lactation also plays an important role in the litter ADG, as sows at similar feed intake levels that lose weight have higher litter ADG and wean heavier litters, compared to sows that gain weight.

## Supplementary Material

txag140_Supplementary_Data

## List of abbreviations

AA: amino acid
ADFI: average daily feed intake
ADG: average daily gain
BW: body weight
NE: net energy
WSI: wean-to-service interval
