# Supplementary material for: Effect of sow feed intake on litter growth and sow body weight change from farrowing to weaning
Source: Transl Anim Sci. 2026 Sep 16;10:txag140. doi: 10.1093/tas/txag140 (PMC13677080; doi:10.1093/tas/txag140)
Supplement: Supplementary file 1 [file txag140_supplementary_data.docx]

| **Supplemental Table 1.** Descriptive statistics by study^1^ | | | | | | | | | | | | | | | | | | | |  |
| --- | --- | --- | --- | --- | --- | --- | --- | --- | --- | --- | --- | --- | --- | --- | --- | --- | --- | --- | --- | --- |
|  |  |  |  | Sow body weight, kg | | |  |  |  | Litter size, n | |  | | Litter weight, kg | |  | | Pig weight, kg | | |
| Study | Gilts, n | Sows, n | Entry | | Farrow | Weaning | | ADFI, kg | d 2 | | Weaning | | d 2 | | Weaning | | d 2 | | Weaning | |
| 1 | 34 | 61 | 256 | | 228 | 226 | | 7.0 | 13.8 | | 13.6 | | 23.5 | | 75.8 | | 1.7 | | 5.6 | |
| 2 | 14 | 42 | 264 | | 243 | 237 | | 7.3 | 14.1 | | 13.6 | | 20.0 | | 78.2 | | 1.8 | | 5.8 | |
| 3 | 313 | 402 | 217 | | 196 | 190 | | 6.9 | 16.7 | | 14.6 | | 24.3 | | 71.5 | | 1.5 | | 4.9 | |
| 4 | 192 | 777 | 234 | | 210 | 208 | | 7.4 | 14.2 | | 13.0 | | 23.5 | | 83.9 | | 1.6 | | 6.4 | |
| 5 | 108 | 404 | 294 | | 266 | 257 | | 6.3 | 13.8 | | 11.7 | | 19.0 | | 66.6 | | 1.4 | | 5.7 | |
| 6 | 135 | 804 | 274 | | 250 | 264 | | 6.2 | 13.7 | | 11.5 | | 20.4 | | 69.7 | | 1.5 | | 6.1 | |
| 7 | 81 | 298 | 281 | | 255 | 267 | | 6.7 | 14.5 | | 12.5 | | 21.8 | | 75.9 | | 1.5 | | 6.1 | |
| 8 | 114 | 605 | 291 | | 264 | 27 | | 8.0 | 14.8 | | 12.5 | | 22.2 | | 80.1 | | 1.5 | | 6.4 | |

^1^ Data from 8 trials, totaling 4.384 sows, 991 gilts and 3,393 multiparous sows.
